# Risk Factors Associated with Avian Influenza Subtype H9 Outbreaks in Poultry Farms of Central Lowland Nepal

**DOI:** 10.3390/idr14040056

**Published:** 2022-07-18

**Authors:** Deepak Subedi, Parshuram Phuyal, Suman Bhandari, Milan Kandel, Shambhu Shah, Gaurav Rawal, Surendra Karki, Santosh Dhakal

**Affiliations:** 1 Paklihawa Campus, Institute of Agriculture and Animal Science (IAAS), Tribhuvan University, Siddharthanagar 32900, Nepal; sumanlfc8@gmail.com (S.B.); shambhushah@gmail.com (S.S.); 2 National Avian Disease Investigation Laboratory, Bharatpur 44200, Nepal; parsuphuyal@gmail.com; 3 Faculty of Animal Science, Veterinary Science and Fisheries (FAVF), Agriculture and Forestry University, Bharatpur 44200, Nepal; milankandel377@gmail.com; 4 Veterinary Diagnostic and Production Animal Medicine, College of Veterinary Medicine, Iowa State University, Ames, IA 50011, USA; grawal@iastate.edu; 5 Food and Agriculture Organization of the UN (FAO), Emergency Center for Transboundary Animal Diseases (ECTAD), Kathmandu 44600, Nepal; 6 W. Harry Feinstone Department of Molecular Microbiology and Immunology, Johns Hopkins Bloomberg School of Public Health, Baltimore, MD 21205, USA

**Keywords:** avian influenza, risk factors, poultry farms, Nepal

## Abstract

Low pathogenic avian influenza (LPAI) of subtype H9 outbreaks have been frequently occurring in major commercial hubs of Nepal including Chitwan, a central lowland area, causing substantial economic losses to the farmers. However, the risk factors associated with these outbreaks have been poorly understood, and hence, this case-control study was conducted in Chitwan, Nawalpur, and Makawanpur districts of Nepal from October 2019 to March 2020. A total of 102 farms were selected in which 51 were case farms, and 51 were controls. Case farms were avian influenza (AI)-subtype-H9-confirmed farms through polymerase chain reaction (PCR) assays on poultry samples. Control farms included farms that were AI-negative in the antigen test brought to the National Avian Disease Investigation Laboratory, Chitwan, for diagnosis during the study period. Each farm was visited to collect information using a semi-structured questionnaire. A total of 25 variables representing farm characteristics and biosecurity measures were considered as potential risk factors. The final multivariable model showed that distance of less than 0.5 km from the main road (OR = 4.04, 95% CI = 1.20–13.56, *p* = 0.023), distance of less than 1 km from a nearest infected farm (OR = 76.42, 95% CI = 7.17–814.06, *p* = 0.0003), and wild birds coming around the farm (OR = 6.12, 95% CI = 1.99–18.79, *p* = 0.0015) were risk factors for avian influenza type H9, whereas using apron or separate cloth inside the shed (OR = 0.109, 95% CI = 0.020–0.577, *p* = 0.0092) was shown to reduce the risk of farms being positive for AI subtype H9. These findings suggest that due consideration should be given to site selection while establishing the farms and the importance of implementing appropriate biosecurity measures, such as using separate cloth inside the shed and preventing the entry of wild birds inside the farm to reduce the potential risk of introduction of avian influenza type H9 to their poultry farms.

## 1. Introduction

Avian influenza virus (AIV) is a segmented negative-sense RNA virus, which belongs to the Orthomyxoviridae family [1,2]. Based on the ability to cause disease in birds, type A influenza virus is broadly divided into the highly pathogenic avian influenza virus (HPAIV), causing up to 100% mortality, and low-pathogenic avian influenza virus (LPAIV), causing much milder, primarily respiratory disease [3]. Although not all viruses of these subtypes cause HPAI, all outbreaks of HPAI to date are caused by either H5 or H7 subtypes [3,4]. Further, the poultry industry is frequently affected by the LPAI, the major causative agent being the H9 subtype. Outbreaks of AI subtype H9 in poultry have been reported in many countries since the mid-1990s and reached to the panzootic proportions [5]. In AIV subtype H9N2, there are two phylogeographic lineages: American and Eurasian lineages [6]. The Eurasian lineage is circulating in Asia, the Middle East, and Europe and is divided into two major lineages, namely Y280 and G1, and one minor Korean lineage [6,7]. Although H9N2 is circulating among wild birds and domestic poultry, it can also occasionally infect pigs and humans [7,8]. At the receptor-binding site of the hemagglutinin (HA) gene of AIV, subtype H9 has human influenza virus-like receptor specificity, demonstrating its zoonotic potential [8]. Cases of avian-to-human transmission of G1-lineage viruses have been confirmed in China, Hong Kong, Bangladesh, and Egypt and Y280-lineage viruses in China [7]. Besides the potential zoonotic threat, LPAI H9 also causes a great economic loss in the poultry industry due to moderate-to-high mortality and decrease in egg production in layers [9,10]. In poultry, outbreaks have been reported from many Asian countries, including China (1994), Pakistan (1994, 2009–2010), Korea (1996), Hong Kong (1997), Iran (1998), United Arab Emirates (2000–2003), Israel (2000–2006), Jordan (2003), Lebanon (2004), Iraq (2004–2007), Japan (2005, 2015–2016), Bangladesh (2016), and Nepal (2018–2019) [5,6,7,11,12,13].

Nepal is an agricultural country where the farming system is mostly subsistence-based, and crops and livestock farming are integrated. Livestock along with fisheries contribute around 12.5% of the total GDP, while the poultry sector alone contributes nearly 4% of total GDP [14]. The Nepal Commercial Poultry Survey, 2014–2015, showed that there are 7368 (35%) commercial poultry farms in Bagmati province and 2993 (14%) in Gandaki province [15], which are the areas of this study. Chitwan district alone has 1920 (8.7%) commercial farms and ranks first nationally in broiler (10%) and egg production (68%), while Makawanpur district ranks second in egg production (5%) [15]. In Nepal, there are two distinct methods of poultry production: scavenging and commercial. The scavenging system is common in rural regions, with the often indigenous chickens of Nepal (Sakini, Ghanti Khuile (naked neck), and Puwakh Ulte) and the Giriraj breed of India. In Chitwan district and its vicinity, which are the study area, there are large number of commercial layers, broilers, and breeders farms, and products from these farms are distributed to different part of the country. The poultry sector provides more than fifty thousand permanent jobs and nearly half a million temporary jobs in Nepal [15]. Since the past three decades, the poultry sector is rapidly commercializing, making it nearly self-sufficient to fulfill the demand in eggs and broiler meat in Nepal [16]. However, there are several challenges in farm management and biosecurity measures implementation, resulting in high average mortality rates of poultry: 12.8% in broilers, 9.2% in layers, and 7.0% in breeders [15].

Outbreaks of avian influenza is always a concern due to its economic impact and public health threat. The first outbreak of HPAI was reported in Nepal on 16th January 2009 in the Jhapa district, the eastern part of Nepal [17], after which it has spread to several districts of the country, causing socioeconomic impacts in the outbreak areas [18]. More than 255 outbreaks of H5N1 and one outbreak of H5N8 have been reported from commercial and backyard poultry farms from different districts, including Chitwan, Nawalpur, and Makawanpur, in Nepal from 2009 to 2019 [19]. During this period, there were deaths of more than 0.2 million poultry from HPAI H5N1, and more than 2 million birds were destroyed to control the spread of disease [19]. The first human casualty from HPAI H5N1 was confirmed in Nepal on 30 April 2019 [12]. Moreover, the circulation of LPAI of the H9 subtype began to be detected in Nepal from 2013–2014. In 2014, influenza A H9N2 (KT085/2014(H9N2)) was detected in one sample of ruddy shelduck out of 1811 environmental fecal samples tested as a part of the Influenza A and its subtype surveillance in wild migratory birds in Nepal [20].

In the poultry population, cases of AI subtype H9 began to surface since 2013 and has been increasing since then. In the samples tested at Central Veterinary Laboratory (CVL) from March 2018 to April 2019, 105 farms tested positive for AI subtype H9 in Kathmandu valley [12]. In Chitwan, the major poultry hub of Nepal, AI subtype H9 was found circulating, which was indicated by the detection of antibodies in ducks (1.08% in 2009 and 10% in 2013–2014) [21,22]. Several outbreaks of AI subtype H9 have been reported in Chitwan, Nawalpur, and Makawanpur districts in chickens since 2013, which has affected the livelihood of farmers and the economy. Although avian influenza has affected Nepalese farmers massively, there is no practice of influenza vaccination, and it is banned in the country.

Different research has assessed risk factors associated with avian influenza outbreaks in various countries of the world [11,12,13,23,24,25,26]. For example, Chaudhry et al. (2015) identified “being near case/infected farms” and “a previous history of the infectious bursal disease (IBD) on the farm” as risk factors of AI subtype H9 in commercial poultry farms in Pakistan [11]. Similarly, “the presence of wild birds on the farms” was a risk factor, and “the presence of a footbath at the entrance of farm” and “changing of gloves” were protective for AI subtype infection in commercial poultry farms of Punjab province and Islamabad Capital Territory, Pakistan [25]. However, very few studies have been conducted to identify the risk factors of AI subtype H9 in Nepal [12]. Gompo et al. (2020) identified “farms that have flock size greater than median flock size of study farms”, “farms that did not apply rules to wear boots for visitors inside the farms”, and “other commercial farms located within one km periphery” as risk factors of AI subtype H9 in Kathmandu Valley, Nepal [12]. No prior research was conducted to evaluate risk factors of avian influenza type H9 in Chitwan, Nawalpur, and Makawanpur districts, which are the major commercial poultry production districts of Nepal. Evaluation and identification of risk factors of AI subtype H9 in major poultry hubs of Nepal would help in designing preventive measures against H9 outbreaks in the future.

## 2. Materials and Methods

### 2.1. Study Design

**Figure 1:**
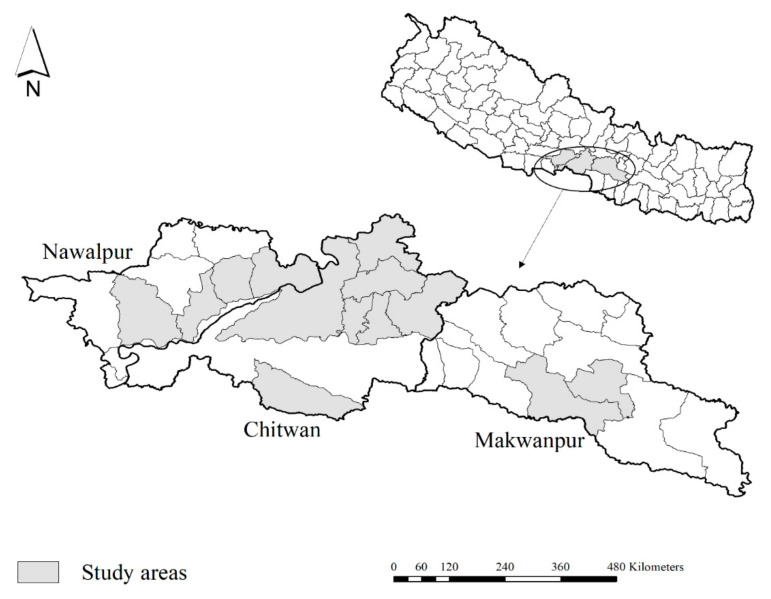
Map of Nepal showing study areas in Chitwan, Nawalpur, and Makwanpur districts. Map was created using ArcGIS version 10.8 (ESRI, West Redlands, CA, USA).

The study population was poultry farms (broilers, layers, backyard, and breeders) from Chitwan, Nawalpur, and Makwanpur districts (Figure 1), which had submitted dead or live birds to National Avian Disease Investigation Laboratory (NADIL), Chitwan, Nepal, for disease diagnosis from October 2019 to March 2020. Farmers usually take their diseased or dead birds in NADIL to identify the cause of problem (mortality and morbidity) in the farm, which may be due to bacteria, viral, fungal, mycoplasmal infection, or management problems, and in general, they send 2–10 birds for the diagnosis of their problem. In the study area, once farmers notice any kind of abnormality in their birds, either they call poultry health workers in their farms, or they send their bird to the only avian laboratory in study area. They usually take both live and dead birds by motorcycle or by private vehicle in a paper box or plastic bags. The duration between infection and laboratory diagnosis could be 1–3 days.

A 1:1 matched case-control study design was used. A case farm was defined as any poultry farm that had submitted a dead or live bird in NADIL, Chitwan, for diagnosis of H9 or any other disease during the study period and was confirmed positive for AI subtype H9 initially by rapid antigen detection test kit (AIV Ag Test kit, Bionote, Gyeonggi-do, Republic of Korea) in NADIL followed by polymerase chain reaction (PCR) in Central Veterinary Laboratory (CVL), Tripureswor, Kathmandu. The H9 V2 TaqMan PCR assay was performed for the confirmed diagnosis of AI subtype H9 [27]. The conserved region in the HA2 subunit of the H9 HA gene was the target gene. H9 Fwd: 5′ATGGGGTTTGCTGCC-3′ and H9 Rev3: 5′-ATATACAAATGTTGCAYCTGCA-3′ were the primers used, and H9 Probe V4: 5′-FAM-TTCTGGGCYATGTCCAATGG-BHQ-1-3′ was used in the PCR assay.

A control farm was defined as any poultry farm in the study area that had submitted dead or live birds in NADIL for diagnosis of any diseases that were confirmed negative by rapid antigen detection test kit and with the same age (week) and type as a case farm.

To achieve 80% power to detect an odds ratio of 3.0 with 95% confidence interval, assuming 40% exposure in the controls, 52 case farms and 52 control farms were required based on Ausvet Epitools [28]. We first selected 52 farms in the case category but later dropped one farm, as a matching control could not be found. Finally, a total of 102 farms (51 cases and 51 controls) were included in the study.

### 2.2. Data Collection

The questionnaire (Appendix A) was pretested among 15 farmers for its validity during their visit to NADIL. A preliminary interview was conducted among farmers after their birds confirmed either positive (case) or negative (control) by rapid antigen detection in NADIL. The study objective was explained to farmers, and the phone number of farmers along with the address of each farm was collected during a preliminary interview. After a confirmed diagnosis of AI subtype H9 by PCR from CVL, Tripureswor, Kathmandu, the case farms were visited, and data were collected via structured interviews using standardized open- and close-ended questions. A similar approach was used to collect the data from control farms. The potential risk factors were selected based on literature review and expert opinions [11,12,13,23,24,25,26]. The risk factors selected were broadly classified into the following sections: (1) farm and flock characteristics and (2) farm management and biosecurity, which are summarized in Table S1.

### 2.3. Statistical Analysis

Data were entered and processed in Microsoft Excel 2016 (Microsoft, Redmond, WA, USA). In total, 25 variables covering farm and flock characteristics and farm management and biosecurity measures were considered for the analysis. Under farm and flock characteristics; median flock size, distance from the main road, distance from the nearest poultry farm, distance from the nearest infected farm, age of the flock during sample submission in the lab, history of infectious bursal disease (IBD) in the flock, history of *E. coli* in the flock, and history of H9 in the farm were considered (Supplementary material, Table S1). Under farm management and biosecurity, fumigation, water source, flooring of the farm, drinking water system, sharing of farm instruments or feed, use of apron or separate cloth inside the shed, use of boots or separate slippers, footbath at the farm entrance, visitors allowed inside poultry shed, spraying workers before entering poultry shed, spraying visitors before entering inside the farm, disinfection around the farm, fully fenced farm, wild and other birds coming around the farm, presence of rodents inside poultry shed, pond or water reservoir around the farm, and farm biosecurity sign were considered (Supplementary material, Table S1). Among these variables, median flock size, distance from the main road, distance from the nearest poultry farm, distance from the nearest infected farm, and age of the flock during sample submission in the lab were continuous, while others were binary. The continuous variables were tested for normality and were found to be non-normal. Therefore, these continuous variables were transfigured into binary categorical variables using medians to avoid the problem of linearity. The ages of birds are categorized into two groups (≤28.7 weeks and >28.7 weeks) with a median age of 28.7 weeks as a cut-off. The flock size was categorized into two groups with a median flock size of 2000 as a cut-off (≤2000 and >2000).

Multicollinearity among the selected 25 variables was tested using Spearman rank correlation. None of the variables correlated by more than 60%. Therefore, all 25 variables were considered for the univariable logistic regression. We first applied univariable logistic regression analysis to assess the association of individual potential risk factors and to screen them for the multivariable model. A cut-off of *p* ≤ 0.15 was used to screen the variables for consideration in the multivariable model [12]. The selected variable from the univariable model was run for a multivariable model using the “PROC LOGISTIC” procedure in SAS 9.4 (SAS Institute Inc, North Carolina, USA) using the backyard logistic regression analysis. A cut-off of *p* < 0.05 was used to include the variable in the final model. Corresponding odds ratio (OR) and their 95% confidence intervals (CI) were calculated to measure the strength of association. The model fit for the final multivariable model was evaluated using the Hosmer–Lemeshow test, which showed that the model fit was good (chi-square value = 3.63; *p*-value = 0.73).

## 3. Results

**Figure 2:**
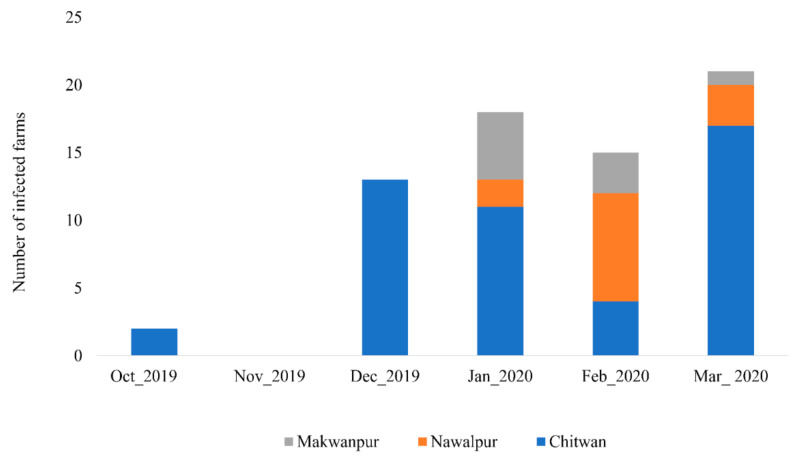
Epidemic curve of avian influenza subtype H9 outbreaks in poultry farms of central lowland Nepal.

During the study period, samples from 74 commercial and backyard farms tested positive for avian influenza in antigen detection kit within the study area. Among them, 69 farms were confirmed positive for AI subtype H9 by PCR, and none were positive for the highly pathogenic subtypes H5 and H7. The epidemic curve of AI subtype H9 outbreaks in poultry farms of the study area from October 2019 to March 2020 is shown in Figure 2. An outbreak started in October 2019 in two farms in the Chitwan district, while there was no outbreak in the study area in November 2019. The highest number of cases was reported in March 2020 with 21 farms infected (Figure 2).

A total of 25 variables were evaluated as potential risk factors and classified into two categories: farm and flock characteristics and farm management and biosecurity. Out of 25 variables, 11 variables that met the criteria of *p* ≤ 0.15 in the univariate analysis were retained for the multivariable analysis. Among them, four variables related to farm and flock characteristic, namely median flock size (OR = 2.417, 95% CI = 1.088–5.368, *p* = 0.0302), distance from the main road (OR = 2.465, 95% CI = 1.052–5.779, *p* = 0.0379), distance from nearest infected farm (OR = 32.258, 95% CI = 4.121–252.523, *p* = 0.0009), and history of IBD in the flock (OR = 4.400, 95% CI = 1.148–16.867, *p* = 0.0307), were significant in the univariate analysis (Table 1). Under farm management and biosecurity, seven variables were retained for multivariable analysis. They include fumigation (OR = 0.492, 95% CI = 0.186–1.302, *p* = 0.1530), sharing of farm instruments or feed (OR = 3.429, 95% CI = 0.871–13.502, *p* = 0.0781), use of apron or separate cloth inside the shed (OR = 0.210, 95% CI = 0.079–0.554, *p* = 0.0016), use of boots or separate slippers (OR = 0.318, 95% CI = 0.119–0.854, *p* = 0.0230), visitors allowed inside poultry shed (OR = 2.810, 95% CI = 1.219–6.480, *p* = 0.0153), wild and other birds coming around the farm (OR = 4.923, 95% CI = 2.109–11.493, *p* = 0.0002), and farm biosecurity sign (OR = 6.125, 95% CI = 1.996–18.792, *p* = 0.0015) (Table 1).

From the final multivariable logistic regression, three variables were identified as the risk factors, and one variable was identified as protective. The risk factors include the distance from the main road, distance from the infected farms, and wild birds coming around the farms, while the protective variable was the use of a separate apron or cloth inside the farm (Table 2). The farms that were ≤0.5 km from the main road were four times more likely to get an infection of AI subtype H9 (OR = 4.043, 95% CI = 1.205–13.564, *p* = 0.0237) compared to farms that are >0.5 km away from the main road. The odds of infection was 76 times higher for the farms that were ≤1 km from the nearest infected farm (OR = 76.420, 95% CI = 7.174–814.064, *p* = 0.0003) than farms that were >1 km away. Likewise, farms where wild birds were seen frequently were six times more likely to be positive for AI subtype H9 infection (OR = 6.125, 95% CI = 1.996–18.792, *p* = 0.0015) (Table 2). The use of an apron or separate cloth inside the shed was protective (OR = 0.109, 95% CI = 0.020–0.577, *p* = 0.0092) against the infection of AI subtype H9 (Table 2).

## 4. Discussion

There are only a few studies being carried out in Nepal to assess risk factors of AI subtype H9. This is the first case-control study, to the best of our knowledge, being conducted in and around the Chitwan district, which is the poultry hub of Nepal, to identify the associated risk factors. In total, 4 variables out of 25 potential risk factors evaluated were found to be associated with AI subtype H9 in central lowland Nepal.

Poultry farms within a distance of 0.5 km from the main road were found four times more likely to be positive for AI subtype H9 compared to farms that were more than 0.5 km away from the main road. This makes biological sense, as different vehicles carrying poultry and poultry products frequently travel in the main road, and there is a high likelihood of farms near the main road becoming infected, as infected particulate materials such as feathers might be brought into the farms through the wind [29]. Our finding is in agreement with the finding of Chaudhry et al. (2015), who found that the “distance of the farm from the main road of 0.5 km” was a risk factor for AI subtype H9N2 in commercial poultry farms in Pakistan [11]. Studies in Vietnam [30], China [31], and Romania [32] also confirmed that closeness to the main road was associated with avian influenza outbreaks.

In this study, farms within 1 km from an infected farm were 76 times at higher risk to acquire AI H9 infection. It is highly likely that when there are other infected farms in the nearby premises, the virus may enter the farm through the movement of people or the sharing of equipment. A similar finding was observed by Chaudhry et al. (2015) in Pakistan, where farms in a distance of less than 1 km from the infected farms were at a 44.4 times higher risk to contract AI subtype H9N2 [11]. Likewise, Nishiguchi et al. (2005) identified distance of 1000–1500 m from the nearest case farm was a risk factor for another AI subtype H5N2 (OR = 20.1) in Japan [13]. Gompo et al. (2020) found that having other farms within 1 km distance increases the risk of farms being positive for H9 [12].

Wild birds coming around the farm was associated with the AI subtype H9 outbreak in this study. Wild birds, which are a potential reservoir of AI viruses, may contaminate the environment via their droppings in and around the poultry farm and contribute to the transmission of AI viruses including AI subtype H9 [5,33]. Chitwan National Park and other community forests are nearby the study area, giving opportunity for wild birds to come near the commercial poultry farms. Other studies have also identified contact with wild birds as a risk factor for the introduction of AI subtype H9 onto farms. For example, the presence of geese on the farm was a risk factor of an avian influenza outbreak in Vietnam [34]. Wang et al. (2013) also identified contact with wild birds as a risk factor for the presence of infectious diseases in backyard poultry [35]. In the study of Biswas et. al (2009), contact of backyard poultry with pigeons was a risk factor for avian influenza infection in commercial farms of Bangladesh [24].

Using a separate apron or cloth within the farm was found to reduce the chances of farms being positive for AI subtype H9 in our study. Viruses might be carried on the clothes of workers of poultry farms. If they use an apron or change clothes while they go inside the farm, it will reduce the chances of pathogens entering the farm through their clothes. Poor biosecurity measures in the farms, including visitors, have also been identified as risk factors for avian influenza outbreaks in poultry farms [12,13,36].

This study includes only farms that were brought to the National Avian Disease Diagnostic Laboratory, Chitwan. The big poultry farms are less likely to bring their birds for diagnosis, as most of them have their own veterinarians and tend to diagnose based on clinical signs and postmortem lesions. Farmers far away from the laboratory are also less likely to bring their samples compared to nearby farms. The other limitations are that the responses provided by the responders might be biased, possibly due to the “recall bias”, and there could have been selection bias due to interval of laboratory diagnosis and questionnaire survey. For case farms, rapid antigen test followed by PCR was used, but for control farms, only rapid antigen test kits were used, and this may have indicated false positives. Another limitation of the study is that a grouping variable, for example, farm, would have been helpful during the analysis to determine how much variation in the survey data was attributable to individual farms and how much is due to specific risk factors.

## 5. Conclusions

Outbreaks of LPAI subtype H9 are a growing concern for poultry farmers in major poultry producing areas of Nepal. Here, we conducted a case-control study in the major poultry farming districts of Nepal to identify the risk factors associated with the outbreaks of AI subtype H9. We identified that having farms closer to the major roads, having infected farms nearby, and having contact with wild birds increase the risk of AI subtype H9 in farms, while using an apron or separate cloth at the farm decreases the chance of farms acquiring AI subtype H9. We suggest that farmers give due consideration to selection of the right location before establishing poultry farms. We also suggest practicing good biosecurity measures on their farms, including avoiding access of wild birds to the farms and using proper measures of personal hygiene, including wearing separate aprons or coveralls inside the farms.

## Figures and Tables

**Table 1:** Result of univariable logistic regression analysis showing significant variables on the cut-off value of *p* ≤ 0.15.

Variables	Category	Cases (n = 51)	Control (n = 51)	Odds Ratio	95% CI	*p*-Value
Farm and flock characteristics						
Median flock size	>2000	29	18	2.417	1.088–5.368	0.0302
	≤2000	22	33			
Distance from the main road	≤0.5 km	22	12	2.465	1.052–5.779	0.0379
	>0.5 km	29	39			
Distance from nearest infected farm	≤1 km	20	1	32.258	4.121–252.523	0.0009
	>1 km	31	50			
History of IBD in the flock	Yes	11	3	4.400	1.148–16.867	0.0307
	No	40	48			
Farm management and biosecurity						
Fumigation	Yes	8	14	0.492	0.186–1.302	0.1530
	No	43	37			
Sharing of farm instruments or feed	Yes	9	3	3.429	0.871–13.502	0.0781
	No	42	48			
Use of apron or separate cloth inside the shed	Yes	7	22	0.210	0.079–0.554	0.0016
	No	44	29			
Use of boots or separate slippers	Yes	34	44	0.318	0.119–0.854	0.0230
	No	17	7			
Visitors allowed inside poultry shed	Yes	25	13	2.810	1.219–6.480	0.0153
	No	26	38			
Wild birds coming around the farm	Yes	32	13	4.923	2.109–11.493	0.0002
	No	19	38			
Farm biosecurity sign	Yes	2	8	0.219	0.044–1.090	0.0636
	No	49	43			

**Table 2:** Results of multivariable logistic regression analysis of risk factors associated with avian influenza subtype H9 outbreaks in poultry farms of central lowland Nepal.

Variables *	Category	Odds Ratio	95% CI	*p*-Value
Farm and flock characteristics				
Distance from the main road	≤0.5 km	4.043	1.205–13.564	0.0237
	>0.5 km			
Distance from nearest infected farm	≤1 km	76.420	7.174–814.064	0.0003
	>1 km			
Farm management and biosecurity				
Use of apron or separate cloth inside the shed	Yes	0.109	0.020–0.577	0.0092
	No			
Wild birds coming around the farm	Yes	6.125	1.996–18.792	0.0015
	No			

* The intercept of the final multivariable model is −1.4046.

## Data Availability

Data will be available on reasonable request.

## Supplementary Materials

The following supporting information can be downloaded at: https://www.mdpi.com/article/10.3390/idr14040056/s1, Table S1: Variables considered as potential risk factors of avian influenza subtype H9 outbreaks in poultry farms of central lowland Nepal.

## Appendix A

Questionnaire for the risk factors associated with avian influenza subtype H9 outbreaks in poultry farms of central lowland Nepal.

I………………………………………………(Participant), understand that I am being asked to participate in survey/questionnaire, and I allow the investigators Subedi to ask the questions related to above mentioned topic for research purpose. I provide my consent to participate in this study and use this information without violating my privacy.

 ………………………

                                                  (Participant

Questionnaire Number:	Case	Control	Date:
Location of the Farm:			

Signature)

**Section 1: Farm and Flock Characteristics**

1. Type of farm: Layers/Broilers/Breeders/Local Backyard/Duck/Giriraj/Quail
2. Capacity of farm: ............................................................................ _________ (no. of birds)
3. Number of poultry shed…………………………………...……………….…. _________
4. Distance of the farm from main road: .............................................................................Km
5. Distance Between Poultry Shed and Road in Km..........................................................Km
6. Distance of farm from nearest poultry farm: ............................................ ________ (Km)
7. Distance from nearest case (infected) farm: ................................________________ (Km)
8. Age of flock during submission: ................................................. _______________ (days)
9. Size of the flock: ............................................................................. _________ (no. of birds)
10. Mortality (%) in the farms ................................................... ____________________ (%)
11. Morbidity numbers in the farm during sample submission ____________________
12. Flock affected by infectious bursal disease ......................................................**Yes** □ **No** □
13. Flock affected by E. Coli infection .................................................................... **Yes** □ **No** □
14. Previous history of H9 outbreak in farm.......................................................... **Yes** □ **No** □

**Section 2: Farm Management**

15. Fumigation in the farm....................................................................................... **Yes** □ **No** □
16. Culling of disease bird........................................................................................ **Yes** □ **No** □
17. Water Supply to birds……...……….…………Well/Boring/Stream/Government Taps
18. Flooring of the farm………………………...……………. Cemented/Muddy/Wooden
19. Feeding system…………………………….…...………………………. Self/Automated
20. Drinking water system………………...…………….…………….….... Self/Automated
21. Number of workers in the farm ………………………………………………………….
22. Farm owners live in the farm ............................................................................ **Yes** □ **No** □
23. Sharing of farm instruments............................................................................... **Yes** □ **No** □
24. Duration of different batch of poultry……………….…………………….………. (Days)

**Section 3: Biosecurity**

25. Use of apron/separate clothes before entering the farm ............................... **Yes** □ **No** □
26. Use of boot/separate slippers before entering the farm ................................. **Yes**□ **No** □
27. Footbath at the farm entrance .............................................................…….…...**Yes**□ **No** □
28. Visitors allowed in the farm................................................................…….......**Yes** □ **No** □
29. Vaccination of H9.......................….…………………………………………......**Yes** □ **No** □
30. Spraying workers before entering into poultry shed .....................................**Yes** □ **No** □
31. Spraying visitors before entering into farm......................................................**Yes** □ **No** □
32. Disinfection around the sheds ...........................................................................**Yes** □ **No** □
33. Is the farm fully fenced? …………………………………………………........**Yes** □ **No** □
34. Presence of rodents inside poultry shed........................................................... **Yes** □ **No** □
35. Wild birds coming in the farm ...........................................................................**Yes** □ **No** □
36. Pond (s)/water reservoir inside/near your farm? ............................................**Yes** □ **No** □
37. Farm bio-security sign………………………………………………………...**Yes** □ **No** □

We thank you for your kind participation.
