# Supplementary material for: Risk Factors Associated with Avian Influenza Subtype H9 Outbreaks in Poultry Farms of Central Lowland Nepal
Source: Infect Dis Rep. 2022 Jul 18;14(4):525–36. doi: 10.3390/idr14040056 (PMC9326661; doi:10.3390/idr14040056)
Supplement: Supplementary file 1 [file idr-14-00056-s001.zip › idr-1759227-supplementary.pdf]

Table S1: Variables considered as potential risk factors of avian influenza subtype H9 outbreaks in poultry farms of central lowland Nepal.

SN	Variables	Category	Cases (n=51)	Control (n=51)
	**Farm and flock characteristics**			
**1**	Median flock size	>2000	29	18
		≤2000	22	33
**2**	Distance from the main road	≤0.5 Km	22	12
		>0.5 Km	29	39
**3**	Distance from the nearest poultry farm	≤1 Km	41	44
		>1 Km	10	7
**4**	Distance from the nearest infected farm	≤1 Km	20	1
		>1 Km	31	50
**5**	Age of the flock during sample submission in the lab	>28.7 weeks	25	24
		≤28.7 Weeks	26	27
**6**	History of IBD in the flock	Yes	11	3
		No	40	48
**7**	History of *E. coli* in the flock	Yes	28	26
		No	23	25
**8**	History of H9 in the farm	Yes	25	0
		No	26	51
	**Farm management and biosecurity**			
**9**	Fumigation	Yes	8	14
		No	43	37
**10**	Water Source	Tap Water	26	21
		Other Source	25	30
**11**	The flooring of the farm	Cemented	47	48
		Other	4	3
**12**	Drinking water system	Automatic	32	27
		Manual	19	24
**13**	Sharing of farm instruments or feed	Yes	9	3
		No	42	48
**14**	Use of apron or separate cloth in the shed	Yes	7	22
		No	44	29
**15**	Use of boot or separate slippers inside the shed	Yes	34	44
		No	17	7
**16**	Footbath at the farm entrance	Yes	5	7
		No	46	44
**17**	Visitors allow inside poultry shed	Yes	25	13
		No	26	38
**18**		Yes	16	22

	Spraying workers before entering poultry shed	No	35	29
**19**	Spraying visitors before entering inside the farm	Yes	30	34
		No	21	17
**20**	Disinfection around the farm	Yes	40	42
		No	11	9
**21**	Farm fully fenced	Yes	16	13
		No	35	38
**22**	Wild birds coming around the farm	Yes	32	13
		No	19	38
**23**	Presence of rodents inside poultry shed	Yes	37	37
		No	14	14
**24**	Pond or water reservoir around the farm	Yes	17	14
		No	37	43
**25**	Biosecurity sign in the farm	Yes	2	8
		No	49	43
